# Innate and Adaptive Anti-SIV Responses in Macaque Semen: Implications for Infectivity and Risk of Transmission

**DOI:** 10.3389/fimmu.2020.00850

**Published:** 2020-05-12

**Authors:** Karunasinee Suphaphiphat, Sibylle Bernard-Stoecklin, Céline Gommet, Benoit Delache, Nathalie Dereuddre-Bosquet, Stephen J. Kent, Bruce D. Wines, P. Mark Hogarth, Roger Le Grand, Mariangela Cavarelli

**Affiliations:** ^1^CEA-Université Paris Sud-INSERM U1184, “Immunology of Viral Infections and Auto-Immune Diseases”, IDMIT Department, IBFJ, Fontenay-aux-Roses, France; ^2^Department of Microbiology and Immunology, Peter Doherty Institute for Infection and Immunity, University of Melbourne, Melbourne, VIC, Australia; ^3^Melbourne Sexual Health Centre and Department of Infectious Diseases, Alfred Hospital and Central Clinical School, Monash University, Melbourne, VIC, Australia; ^4^ARC Centre for Excellence in Convergent Bio-Nano Science and Technology, University of Melbourne, Parkville, VIC, Australia; ^5^Immune Therapies Group, Burnet Institute, Melbourne, VIC, Australia; ^6^Department of Clinical Pathology, University of Melbourne, Melbourne, VIC, Australia; ^7^Department of Immunology and Pathology, Monash University, Melbourne, VIC, Australia

**Keywords:** SIV, cytokines, antibodies, ADCC, CD8^+^ T cells

## Abstract

HIV-1 infection is transmitted primarily by sexual exposure, with semen being the principal contaminated fluid. However, HIV-specific immune response in semen has been understudied. We investigated specific parameters of the innate, cellular, and humoral immune response that may affect semen infectivity in macaques infected with SIVmac251. Serial semen levels of cytokines and chemokines, SIV-specific antibodies, neutralization, and FcγR-mediated functions and SIV-specific T-cell responses were assessed and compared to systemic responses across 53 cynomolgus macaques. SIV infection induced an overall inflammatory state in the semen. Several pro-inflammatory molecules correlated with SIV virus levels. Effector CD8^+^ T cells were expanded in semen upon infection. SIV-specific CD8^+^ T-cells that expressed multiple effector molecules (IFN-γ^+^MIP-1β^+^TNF^+/−^) were induced in the semen of a subset of SIV-infected macaques, but this did not correlate with local viral control. SIV-specific IgG, commonly capable of engaging the FcγRIIIa receptor, was detected in most semen samples although this positively correlated with seminal viral load. Several inflammatory immune responses in semen develop in the context of higher levels of SIV seminal plasma viremia. These inflammatory immune responses could play a role in viral transmission and should be considered in the development of preventive and prophylactic vaccines.

## Introduction

More than 80% of new HIV-1 infections worldwide occur during sexual intercourse, involving mucosal transmission of the virus (1–3). Mucosal and genital tract tissues constitute the major source of HIV-1 contaminating fluids. Semen, which contains both cell-free particles and infected cells (4–6), represents the main vector of HIV-1 dissemination, illustrated in part by transmission occurring more frequently from men to men and women than women to men (7). Transmission of HIV-1 has been correlated with seminal viral load (SVL) and stage of infection (8, 9), but the mechanisms of transmission have not been fully addressed. To establish an infection via the genital mucosa, HIV-1 in semen must overcome substantial immunological and physiological barriers to cross the epithelium and infect underlying target cells. Several factors are known to affect semen infectivity and enhance the risk of HIV-1 transmission to a partner, such as concomitant genital infections and inflammation, natural antimicrobial molecules (β-defensins, secretory leukocytes peptidase inhibitor –SLPI-, lysozyme, lactoferrin), semen-derived amyloid fibrils (10, 11), cytokines (IL-7) (12), and male circumcision (13–17). However, very little is known about the actors of the innate and HIV-specific adaptive immune response present in semen that may limit or enhance viral transmission. Indeed, most studies on anti-HIV-1 responses in the genital tract have focused on women, whereas knowledge about male genital tract (MGT) immunity against HIV-1 is still limited. Improved knowledge of the role of antibodies and T cells present in semen may help in the development of more pertinent strategies in the field of HIV-1 transmission prevention.

Although the role of cell-mediated immunity in reducing HIV-1 replication and killing infected cells has been fully established (18), very few studies have assessed the presence of HIV-specific CD8^+^ T cells and their functionality in semen (19–22). Sheth et al. reported that HIV-specific CD8^+^ T cells in semen do not correlate with reduced local virus shedding (23). However, only a subset of HIV-specific CD8^+^ T cells (polyfunctional cells) are most important in controlling viremia (24) and, it is still unclear whether the presence of HIV-specific polyfunctional cytotoxic cells in semen have an impact on the local shedding of viral particles or infected cells and HIV-1 transmission.

HIV-specific antibodies (Abs) may prevent or partially control mucosal HIV-1/SIV entry (25, 26). In macaques, systemic and mucosal application of neutralizing IgG prevents infection after intravenous or vaginal inoculation of SHIV (27–30). HIV-specific non-neutralizing Abs may also play a protective role through antibody-dependent cell-mediated cytotoxicity (ADCC), induction of viral aggregation, or prevention of viral uptake (31–34). HIV/SIV-specific Abs, mostly IgG, have been reported to be present in abundant quantities and at high frequencies (81–100%) in the semen of HIV^+^ men and SIV^+^ macaques (26, 35, 36). However, their ability to neutralize the virus and induce ADCC has been little investigated (37, 38).

Here, we focused on the innate and adaptive immune response in the semen of SIV-infected macaques that we used as a model of HIV-1 infection and AIDS. We defined the dynamics of SVL, cytokine levels, the SIV-specific Ab response, and CD8^+^ T cell levels during acute and chronic infection. An understanding of the humoral and cellular immune function in semen and its effect on local viral replication in the cynomolgus macaque model should provide a framework for developing immunological interventions to prevent sexual transmission of HIV-1.

## Materials and Methods

### Ethical Statement and Animals Care

Cynomolgus macaques (*Macaca fascicularis*), weighing 5–11 kg, were imported from Mauritius and housed according to European guidelines for animal care (Journal Officiel des Communautés Européennes, L 358, December 18, 1986 and new directive 63/2010). All work related to animals was conducted in compliance with institutional guidelines and protocols approved by the local ethics committee (Comité d'Ethique en Experimentation Animale de la Direction des Sciences du Vivant au CEA under numbers: 2015032511332650(APAFIS#373); 10-062; 12-103).

### Infection and Sample Collection

Animals were tested negative for antibody response to SIV, simian retrovirus type D (SRV), or simian T-cell lymphotropic virus (STLV) at the beginning of the study. Adult males were infected by intrarectal or intravenous inoculation of 50 or 5,000 animal infectious doses 50% (AID50) respectively, of SIVmac251 isolate (39). Semen and blood were collected from sedated animals following 5 mg/kg intra-muscular injection of Zoletil®100 (Virbac, Carros, France).

Electroejaculation was performed by intrarectal electrostimulation of the prostate using a probe (12.7 mm diameter) lubricated with a conductor gel and an AC-1 electro-ejaculator (Beltron Instrument, Longmont, USA), as previously described (40). Sequential stimulation was performed with a pattern of six cycles, nine stimulations of 2 s for each cycle, followed by a tenth stimulation lasting 10 s. The voltage was increased every two cycles (1–3 V for the first two, 2–4 V volts for series 3–4, 6–8 V for series 5–6). If a complete ejaculum was not obtained after six cycles of stimulation, a seventh cycle at 7–10 V was performed. Complete ejaculum (volume ranging between 0.1 and 0.5 ml) was immediately diluted in 1.2 ml phosphate buffer saline (PBS) and centrifuged.

Blood samples were collected into BD Vacutainer® Plus Plastic K_3_EDTA tubes for plasma viral load quantification and BD Vacutainer® Plus Plastic SST tubes for serum preparation (BD Biosciences, Le Pont de Claix, France).

### Seminal Plasma and Cell Preparation

Seminal plasma samples were isolated from total semen immediately after collection by spinning 15 min at 775 x g. After separation from seminal plasma, seminal cells were diluted in 14 ml complete medium, consisting of RPMI-1640 Glutamax medium (Invitrogen, Carlsbad, USA) supplemented with a mixture of penicillin, streptomycin, and neomycin (Invitrogen) and 10% FCS (Lonza, Allendale, USA), and kept at room temperature for a maximum of 1 h. Cells were then centrifuged 10 min at 1,500 × g, filtered through a 70-μM sieve and washed with 5 ml PBS supplemented with 10% FCS.

### Quantification of Blood and Semen RNA Viral Load

Blood plasma was isolated from EDTA blood samples by centrifugation for 10 min at 1,500 × g and cryopreserved at −80°C. Seminal plasma was maintained on ice for a maximum of 1 h and cryopreserved at −80°C. Blood viral RNA was prepared from 250 μL cell-free plasma using the Nucleospin 96 RNA kit (Macherey Nagel GmbH&Co KG, Düren, Germany), according to the manufacturer's instructions. Retro-transcription and cDNA amplification and quantification were performed in duplicate by RT-qPCR using the Superscript III Platinum one-step quantitative RT-PCR system (Invitrogen, Carlsbad, USA). RT-PCR was performed as previously described (39). The quantification limit (QL) was estimated to be 111 copies/ml and the detection limit (DL) 37 copies/ml. Semen viral RNA was prepared from 500 μL seminal plasma using the QIAamp Ultrasens Virus kit (Qiagen, Courtaboeuf, France), according to the manufacturer's instructions. Quantitative RT-PCR was performed under the same conditions as above, with a QL of 37 copies/ml and a DL of 12.3 copies/ml.

### Cytokine Quantification in Semen

The concentration of IL-1β, IL-1RA, IL-2, IL-4, IL-5, IL-6, IL-8, IL-10, IL-12/23, IL-13, IL-15, IL-17, IL-18, sCD40L, GM-CSF, G-CSF, TGFα, IFNγ, MIP-1α, MIP-1β, MCP-1, TNF, and VEGF were measured in seminal plasma using the Milliplex® Map Non-Human Primate Cytokine Magnetic Bead Panel—Premixed 23-plex (Merck Millipore, Darmstadt, Germany). Levels of RANTES and TGF-β1, 2, and 3 were measured using monoplex and a 3-plex Milliplex kits. Assays were performed in duplicate using 25 μL seminal fluid. CXCL10/IP-10 was quantified using Quantikine ELISA human IP-10 immunoassay kit (R&D Systems, MN, USA). Samples were thawed at room temperature and centrifuged for 10 min at 1,500 × g to remove cellular components and debris. Immunoassays were performed according to the manufacturer's instructions. Data were acquired using a Bio-Plex 200 instrument and analyzed using Bio-Plex Manager Software, version 6.1 (Bio-Rad, Hercules, USA).

### Phenotypic Characterization of Semen Leukocytes

Staining was performed in parallel on whole blood samples. All staining, except that of whole blood assays, were performed after saturation of Fc receptors in healthy macaque serum (in-house production) for 1 h at 4°C. Amine-reactive dye Live/dead® Fixable Blue (Life Technologies) was used to assess cell viability and exclude dead cells from the analysis. Cells were stained with monoclonal Abs for 30 min at 4°C, washed in PBS/10% FCS and fixed in CellFIX^TM^ (BD Biosciences). Three different antibody panels were used. Panel 1, targeting semen leukocyte subpopulations and T-cell activation, consisted of anti-CD45 PerCp (clone B058-1283), anti-CD3 V500 (clone SP34-2), anti-CD4 PE-Cy7 (clone L200), anti-CD11b Alexa Fluor 700 (clone ICRF44), anti-HLA-DR APC-H7 (clone G46-6), anti-CD69 FITC (clone FN50), and anti-CD95 APC (clone DX2) (all BD Biosciences, Franklin Lakes, USA); anti-CD8 V450 (clone BW138/80; Miltenyi Biotec GmbH, Bergisch Gladbach Germany); and anti-CD28 (clone 25-0289-73; CliniSciences, Nanterre, France). Panel 2, targeting CCR5 and CXCR3 expression on T cells, consisted of the same Abs used in panel 1 plus anti-CCR5 APC and anti-CXCR3 FITC (IgG1, clone 1C6) (both BD Biosciences). Panel 3, targeting LFA-1 and Mac-1 expression on T cells, consisted of the same Abs used in panel 1 plus anti-CD11a PE (IgG1, clone HI111) and anti-CD18 APC (IgG1, clone 6.7) (both BD Biosciences). Corresponding isotype controls of CCR5, CXCR3 CD11a, and CD18 were used at the same concentrations as the reference antibody. Acquisition was performed on a BD LSRII equipped with four lasers (355, 405, 488 and 633 nm) and analyzed using Flowjo v7.6 (Tree Star, Ashland, OR).

### *Ex vivo* Stimulation and Intracellular CD45RA, IFN-γ, TNF, MIP-1β, and IL-2 Staining of Peripheral Blood and Semen Mononuclear Cells

Briefly, 1 × 10^6^ peripheral blood mononuclear cells (PBMCs) were incubated for 1 h at 37°C in 5% CO2 with medium alone, staphylococcus enterotoxin B (2 μg/100 μL), or a commercial pool of SIV gag peptides (89 peptides from p15 and p27, ProteoGenix, Schiltigheim, France) at a concentration of 0.2 μg/100 μL, in the presence of the co-stimulatory Abs CD28 (clone L293, IgG1) and CD49d (clone L25, IgG2b). Semen cells were split into two vials and incubated for 1 h with medium alone or the SIV gag peptide pool/co-stimulatory Abs. Brefeldin A (BD Biosciences) was then added (1 μg/100 μL) and the samples were incubated for 4 h, permeabilized, and stained with combinations of anti-CD45-PerCp (clone D058-1283, IgG1), anti-CD3-APC-Cy7 (clone SP34-2, IgG1), anti-CD8-V500 (clone RPA-T8, IgG1), anti-CD45RA-PE-Cy7 (clone L48, IgG1), anti-CD154-FITC (clone TRAP1, IgG1), anti-IL-2-APC (MQ1-17H12, IgG2a), anti-MIP-1β-PE (clone D21-1351, IgG1), anti-TNF Alexa Fluor 700 (clone Mab11, IgG1), and anti-IFN-γ-V450 (clone B27, IgG1). All Abs used in this panel were from BD Bioscience. A positive response by PBMCs was considered to be SIV-specific if: (1) the response by Gag-stimulated cells was at least 2-fold higher than that of the unstimulated control and (2) the frequency of the CD8^+^ SIV-specific response was > 0.1%. A positive response by semen was considered to be specific if: (1) more than 500 CD8^+^ T cells were acquired and (2) the frequency of positive cells was > 1%.

### Quantification of SIV-Specific IgG Titers in Blood and Semen

Blood serum was isolated from SST blood samples by centrifugation for 10 min at 1,500 × g and cryopreserved at −80°C. Seminal plasma was isolated as described above. ELISA plates (MaxiSorp plates; Nalgene Nunc, Rochester, NY) were coated overnight at 4°C with SIVmac251 gp130 recombinant protein (NIBSC, England) diluted to 1 μg/ml. Wells were washed with wash buffer (PBS 0.05% Tween 20) (Sigma Aldrich) and blocked for 1 h at 37°C with PBS containing 1 mM EDTA and 3% BSA (both from Sigma- Aldrich). Plates were washed five times and incubated with 2-fold serial dilutions of serum and seminal plasma diluted in 3% BSA, starting at 1/200 for serum and 1/20 for seminal plasma. Serum and seminal plasma from the same macaques before infection were used as negative control, whereas a reference positive serum from a SIVmac251-infected cynomolgus macaque was used as positive control. Plates were then washed 5 times and 1/20,000 horseradish peroxidase (HRP)-conjugated goat-anti monkey H+L chain IgG antibody (AbSerotec) was added and incubated for 1 h at 37°C. After washing, the color was developed using 3,3', 5,5-tetramethylbenzidine (TMB) (Life Technologies), followed by the addition of 1 M HCl stop solution. Absorbance at a wavelength of 450 nm was recorded (Tecan SPARK 10M). Antibody titers were calculated by extrapolation from the absorbance as a function of a serum or seminal plasma dilution curve (five-parametric logistic curve) and were defined as the dilution of the test serum or seminal plasma reaching 5 fold the absorbance of the corresponding negative control (serum or seminal plasma taken before infection) tested at 1/200 or 1/20 for serum and seminal plasma, respectively.

### TZM-bl Neutralization Assay

Viral titrations were performed in TZM-bl cells as previously described (41). We used a cut-off value of 2.5-times the background relative luminescence units (RLUs) when quantifying positive infections in the TCID assays, according to the guidelines for the TZM-bl assay. The TCID_50_ was defined as the reciprocal of the viral dilution resulting in 50% positive wells (Reed-Muench calculation). A standard inoculum, corresponding to a virus dilution that yields ~300,000–500,000 RLU equivalents (+/– 15,000 RLUs), was used for the neutralization assay to minimize virus-induced cytopathic effects while maintaining the ability to measure a 2-log reduction in virus infectivity. Both plasma and seminal plasma samples were heat-inactivated at 56°C for 30 min before use in the neutralization assay. Samples were prepared by serial 2-fold dilution, starting from a concentration of 1/40 and 1/160 for plasma and seminal plasma, respectively. The diluted samples were incubated with viral supernatant (SIVmac251) for 1 h. Then 10,000 TZM-bl cells were added to each well. The plate was incubated for 48 h and the luciferase activity measured. Infections were carried out in culture medium containing 15 μg/ml DEAE dextran (diethylaminoethyl; Amersham Biosciences, Fairfield, Connecticut, USA). Additionally, we used HIV-1-based vesicular stomatitis envelope glycoprotein G (VSV-G)-pseudotyped virus as a control for specificity of the neutralization activity. The viral vector expressing luciferase was constructed by substituting the *nef* gene sequence of the HIV-1_NL4−3_ genome with the firefly luciferase gene (42). A similar neutralization assay protocol was used for the experiment with the VSV-G pseudotyped virus as that used for the experiment with the SIVmac251 virus, but in the absence of DEAE dextran. The viral input was 8.75 ng/ml, yielding a comparable RLU value to that obtained with SIV mac251. The 50% neutralization titers (IC_50_) are the dilution of plasma or seminal plasma at which RLUs are reduced by 50% relative to the RLUs in the virus control wells after subtraction of the background RLUs in the control wells with only cells. By analogy, the IC_75_ and IC_90_ were defined as the concentration of Abs able to decrease the percentage of infected cells by 75 and 90%, respectively. The IC_50_, IC_75_, and IC_90_ were calculated using a linear interpolation method (43). For calculating the mean, a value of greater than the highest Ab concentration used was recorded if 50% inhibition was not achieved. In contrast, if 50% inhibition was not achieved at the lowest Ab concentration used, a 2-fold lower concentration than that value was recorded.

### ELISA-Based FcγRIIIa Dimer-Binding Assay

The protocol of the ELISA-based IgG assay using a recombinant, soluble FcγR dimer has been previously described (44). Briefly, ELISA plates (MaxiSorp plates; Nalgene Nunc, Rochester, NY) were coated overnight at 4°C with SIVmac251 gp130 recombinant protein (NIBSC, England) diluted to 1 or 5 μg/ml in PBS (to test blood plasma and seminal plasma Abs, respectively), as well as no Ag as a negative control. HIVIG (#3957; National Institutes of Health AIDS Reagent) was used at 5 μg/ml to normalize the results across all plates. Coated plates were subsequently washed with PBS containing 0.05% Tween 20 (Sigma Aldrich) and blocked with blocking buffer, consisting of PBS containing 1 mM EDTA and 1% BSA (both from Sigma- Aldrich), for 1 h at 37°C. SIV^+^ serum and seminal plasma samples were diluted 1/100 in blocking buffer and incubated in the gp130-coated wells for 1 h at 37°C. Then, purified dimeric macaque rsFcγRIIIa-biotin (I158 allele) was added to the plate at a concentration of 0.1 μg/ml and the plates incubated for 1 h at 37°C. Horseradish peroxidase (HRP)-conjugated streptavidin (Thermo Fisher Scientific) was added and the plates incubated for another 1 h at 37°C. After washing, the color was developed using 3,3', 5,5-tetramethylbenzidine (TMB) (Life Technologies), followed by the addition of 1 M HCl stop solution. Absorbance at a wavelength of 450 nm was recorded. The no-Ag values were subtracted from each Ag sample and the resulting values normalized to 5 μg/ml HIVIG. Final absorbance values were multiplied by the dilution factor (1/100) of each sample. A positive signal was defined as an absorbance value higher than the mean + 3 × SD of the value obtained using sera and seminal plasma from macaques before infection.

### Data Visualization and Statistical Analysis

All data visualization and statistical analysis were carried out using GraphPad Prism 8.1 software (GraphPad software, La Jolla, USA), except for the pie chart of the SIV-specific CD8 T-cell response, which was generated using Spice software (NIH, Bethesda, USA). The non-parametric Spearman rank correlation test was used to investigate the relationship between parameters. The non-parametric Mann-Whitney test was used to compare different groups of macaques and the non-parametric Wilcoxon rank sum test and paired *t*-test were used to compare data from the same macaques at various time points before and after SIV infection. All the tests were two-tailed and *p* values of 0.05 or lower were considered significant, ^*^p < 0.05, ^**^p < 0.01, ^***^p < 0.001, ^****^p < 0.0001.

## Results

### SIV Infection Induces Pro-inflammatory and Immunoregulatory Cytokines in Semen

Seminal plasma levels of inflammatory cytokines may have a major impact on shedding of cell-free and cell-associated virus, and they may affect the state of the recipient's cells in the female reproductive tract (FRT) and rectum, increasing the risk of mucosal HIV-1 transmission. We measured these parameters in seminal plasma samples of macaques intravenously infected with the high dose of 5,000 animal infectious dose 50% (AID_50_) of SIVmac251 virus to define the relationship between cytokine composition and viral seeding of the MGT.

**Figure 1:**
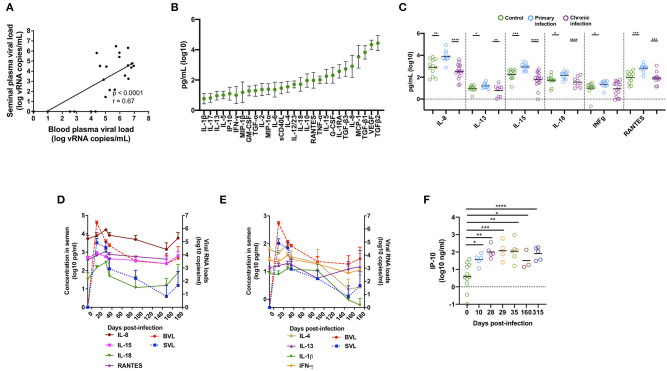
Cytokines and chemokines affected by SIV infection in macaques. **(A)** Spearman correlation between SIV viral load in blood and semen. The analysis was performed on a total of 25 different macaques [*n* = 6 at 10 dpi, *n* = 4 at 14 dpi, and *n* = 15 at the chronic stage (1 year post-infection)]. Three out of 15 macaques at the chronic stage were aviremic in both compartments, whereas four macaques had virus in the blood but not in the semen. **(B)** Mean and standard deviation (SD) of 28 cytokine and chemokine levels in the seminal plasma of 12 uninfected macaques. **(C)** Mean of five pro-inflammatory molecules and the immunoregulatory cytokine IL-13 in SIV^−^ macaques (*n* = 12, green dots) and SIV^+^ macaques at the primary stage (10 or 14 dpi, *n* = 10, blue dots), and the chronic stage (*n* = 16, purple dots). **(D,E)** Longitudinal follow-up of seminal plasma levels of cytokines and chemokines and BVL (red) and SVL (blue) (both are represented by dotted lines) in SIV-infected macaques (*n* = 10 at baseline and at 14 dpi, seven at 28, 35, and 87 dpi, and six at 148 and 171 dpi). The mean and SEM are shown for each time point. **(F)** Mean of IP-10 concentrations in SIV^−^ macaques (*n* = 19) and SIV^+^ macaques at 10 (*n* = 6), 28 (*n* = 6), 29 (*n* = 4), 35 dpi (*n* = 7), 160 (*n* = 3), and 315 (*n* = 5). Significant differences between groups are shown (Mann-Whitney test, *p < 0.05, **p < 0.01, ***p < 0.001, and ****p < 0.0001).

Blood viral load (BVL) strongly correlated with seminal viral load (SVL) (*r* = 0.67, *p* < 0.0001, *n* = 25, Figure 1A), confirming our previously published results (40), as well as those reported for HIV-1-infected patients (6, 45–47). Viral load peaked at 10 days post infection (dpi), followed by a rapid decrease and stabilization in both compartments at three months post-infection (Figures 1D,E).

We measured the seminal plasma levels of 28 cytokines and chemokines in a cross-sectional study on three independent groups of macaques at various stages of infection: before infection (*n* = 12), acute infection (10–14 dpi, *n* = 10), and chronic infection (> 1 year pi, *n* = 16). VEGF, MCP-1, and IL-8 and the active form of TGF-β1-3, were present at high levels (mean > 1,000 pg/ml) in the seminal plasma of uninfected macaques (Figure 1B). The levels of several pro-inflammatory cytokines (IL-8, IL-15, IL-18, IFN- γ, and RANTES) and the immunomodulatory molecule IL-13 were significantly higher in the seminal plasma of macaques during the primary infection (Mann-Whitney test, *p* = 0.001, 0.0006, 0.01, 0.02, 0.0002, and 0.03, respectively) than that of uninfected macaques (Figure 1C and Table 1). These observations were confirmed in 10 macaques longitudinally followed until 170 dpi (Figures 1D,E). Cytokine levels were elevated at 10–14 dpi, with a peak at 28 dpi, followed by a reduction. We observed the same profile for IL-4 and IL-1β. Accordingly, seminal plasma levels of IL-1β, IL-4, IL-8, IL-15, IL-18, RANTES, and TGF-β1, 2 and 3 positively predicted SVL and BVL (Table 1). IL-13 predicted SVL but not BVL, whereas IFN- γ levels did not predict viral shedding in both compartments (Table 1). Interestingly, semen concentration of IP-10 steadily increased during the first month of infection and levels were not stabilized even in the chronic phase (Mann-Whitney test, *p* = 0.01, 0.003, 0.0005, 0.002, 0.02 and < 0.0001 for 10, 28, 29, 35, 160 and 315 dpi, respectively, Figure 1F), suggesting a role for IP-10 in lymphocytes trafficking to mucosal tissues during HIV/SIV infection.

**Table 1:** Seminal levels of cytokines and chemokines affected by SIV infection.

**Molecules**	**No infection** **(Mean (pg/ml) ± SD)**	**Primary infection (Mean (pg/ml) ± SD)**	**Chronic infection (Mean (pg/ml) ± SD)**	**Correlation with BVL**		**Correlation with SVL**	
				***P* value**	***r* value**	***P* value**	***r* value**
IL-1β	4 ± 4	13 ± 20	1 ± 2	0.009	0.62	0.0001	0.71
IL-4	39 ± 35	57 ± 39	7 ± 16	< 0.0001	0.73	0.0003	0.69
IL-8	2,066 ± 2,392	16,271 ± 25,874	806 ± 1,097	< 0.0001	0.71	< 0.0001	0.75
IL-13	10 ± 6	19 ± 11	5 ± 9	0.016	0.48	0.003	0.59
IL-15	255 ± 173	1,073 ± 7,467	131 ± 1,450	< 0.0001	0.75	0.006	0.55
IL-18	78 ± 76	185 ± 114	31 ± 50	< 0.0001	0.72	< 0.0001	0.82
IFNγ	14 ± 8	27 ± 14	18 ± 19	0.37	0.19	0.78	0.06
RANTES	158 ± 147	863 ± 640	164 ± 303	0.0006	0.67	0.002	0.63
TGF-β1	7,571 ± 7,924	19,544 ± 23,463	2,144 ± 4,403	0.003	0.70	0.008	0.69
TGF-β2	43,017 ± 39,647	40,763 ± 32,440	6,768 ± 8,162	0.02	0.66	0.04	0.56
TGF-β3	828 ± 813	1,352 ± 964	291 ± 154	0.005	0.71	0.03	0.58

*Levels of cytokines/chemokines in the seminal plasma of uninfected macaques (n = 12) and during primary (10–14 dpi, n = 10) and chronic (> 1-year pi, n = 16) infection. The correlation with blood viral load (BVL) and seminal viral load (SVL) is shown*.

In conclusion, SIV infection was associated with inflammation in seminal plasma, especially during the first weeks of infection, with pro-inflammatory molecule levels correlating with seminal viral shedding.

### Semen CD8^+^ T Cells Have a Mucosal-Like Phenotype and Are Highly Activated Following SIV Infection

**Figure 2:**
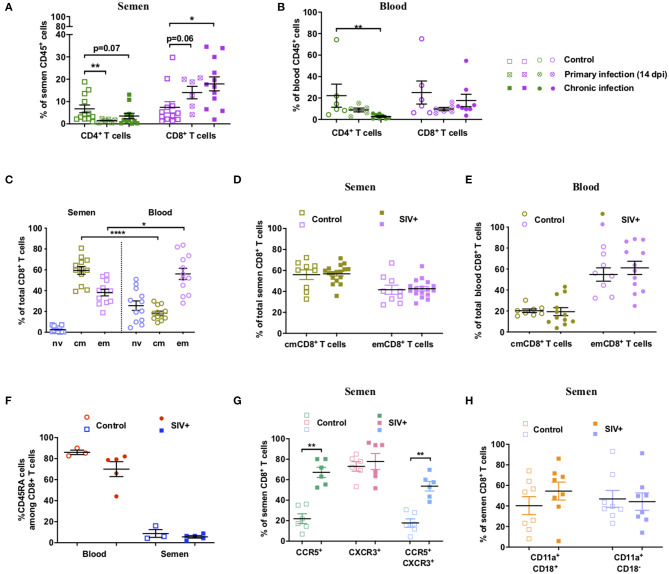
Dynamics and phenotype of semen CD8^+^ T cells and comparison with blood. **(A,B)** Percentage of CD4^+^ and CD8^+^ T cells of total CD45^+^ cells in semen **(A)** and peripheral blood **(B)** in uninfected (*n* = 12 or 6) and SIV^+^ macaques at 14 dpi (*n* = 6) and at the chronic stage (> 1 year pi, *n* = 12 or 8). The mean and SEM is shown. **(C)** Percentage of Tnv (orchid), Tcm (green) and Tem (lavender) CD8^+^ T cells in blood (round dots) and semen (squared dots) of 12 uninfected macaques for which paired blood and semen samples were available. **(D,E)** Percentage of each CD8^+^ T cell subset in the semen **(D)** and blood **(E)** of SIV^−^ (open symbols, *n* = 9 or 8) and chronic SIV^+^ macaques (filled symbols, *n* = 17 or 12). Different control animals were used in panels **(C–E)**. **(F)** CD45RA expression of semen CD8^+^ T cells: percentage of CD45RA^+^CD8^+^ T cells in the blood (red circles) and semen (blue squares) of SIV^−^ (open symbols, *n* = 3) and SIV^+^ (filled symbols, *n* = 5) macaques is shown. **(G)** CCR5 and CXCR3 expression on semen CD8^+^ T cells. Percentage of CCR5^+^ (green squares), CXCR3^+^ (rose squares), and CCR5^+^ CXCR3^+^ (blue squares) cells in SIV^−^ (open squares, *n* = 6) and SIV^+^ (filled squares, *n* = 6) macaques. **(H)** LFA-1 integrin (CD11a^+^CD18^+^) expression on semen CD8^+^ T cells. % of LFA-1^+^ (orange squares) and CD11a^+^CD18^−^ cells (violet squares) in SIV^−^ (open squares, *n* = 8) and SIV^+^ (filled squares, *n* = 8) macaques. The mean and SEM are shown. *indicates significant differences between groups (Mann-Whitney test, *p < 0.05, **p < 0.01, and ****p < 0.0001).

We then performed an in-depth characterization of the phenotype and dynamics of T-cell populations in blood and seminal plasma during SIV infection. The proportion of semen T cells was strongly affected by SIV infection, with rapid, significant, and durable depletion of CD4^+^ T cells (*p* = 0.005 at 14 dpi, Mann-Whitney test), which was paralleled by an increase in CD8^+^ T cells among total CD45^+^ leucocytes (Figure 2A). As expected, CD4^+^ T-cell depletion also occurred in peripheral blood (Figure 2B). These data confirm that macaques infected with SIVmac251 recapitulate the observations reported for HIV-1^+^ patients (35).

Most of the cytokines which showed higher levels in the seminal plasma of SIV^+^ macaques are known to induce T-cell activation. We thus phenotyped the semen CD8 T cells.

In macaques, CD95 and CD28 are commonly used in flow cytometry analysis to discriminate naïve (Tnv, CD95^−^CD28^+^) from central memory (Tcm, CD95^+^CD28^+^) and effector memory (Tem, CD95^+^CD28^−^) T cells (40). In uninfected macaques, semen CD8^+^ T cells were all of the memory phenotype (CD95^+^), with a mean of 59.4% ± 12.9 Tcm cells, 38.2% ± 11.2 Tem cells and 2.4% ± 2.9 Tnv, whereas in the blood naïve cells represented 25.6% ± 15.9 of total CD8^+^ T cells (Figure 2C). The proportion of Tcm cells was higher in the semen than blood, whereas the proportion of Tem cells was slightly higher in the blood (Mann Whitney test, *p* < 0.0001 and 0.017 respectively, for Tcm and Tem) (Figure 2C). There was no difference between non-infected and SIV^+^ macaques in the proportion of each CD8^+^ T cell subset for either seminal plasma (Figure 2D) or blood (Figure 2E).

The expression of CD45RA by memory cells is considered to be a marker of end stage differentiation, as reviewed in (48). We observed a lower proportion of CD45RA^+^ CD8^+^ T cells in the semen than blood of uninfected macaques (mean of 8.8 ± 6.4 vs. 86.0 ± 3.7, Figure 2F). In SIV^+^ macaques, the proportion of semen CD45RA^+^ cells did not significantly change in either compartment (mean of 5.6% ± 2.6 and 70.1% ± 31.1, Figure 2F).

We then analyzed the expression of the chemokine receptors CCR5 and CXCR3, which are effector cell markers. Cells expressing such markers may originate from the MGT rather than the blood. CCR5^+^CXCR3^+^ T cells have been associated with IFN-γ production and a Tc1 profile (49, 50). Moreover, these molecules likely facilitate the highly-directed migration of effector T cells to tissues expressing a gradient of their ligand(s), such as cervico-vaginal tissues and the gut mucosa (51–53). At steady state, 22.0% ± 11.7 of semen CD8^+^ T cells were CCR5^+^CXCR3^−^, 73.0% ± 11.2 CCR5^−^CXCR3^+^, and 17.8% ± 9.9 CCR5^+^CXCR3^+^ (Figure 2G). SIV infection induced a significant increase in the proportion of CCR5^+^CXCR3^−^ and CCR5^+^ CXCR3^+^ cells (Mann-Whitney test, *p* = 0.002 for both subsets, mean of 67.2% ± 12.1 CCR5^+^CXCR3 and 53.7% ± 11.4 CCR5^+^CXCR3^+^ cells; Figure 2G). This suggests that most semen CD8^+^ T cells in SIV^+^ macaques display a Tc1 profile. This is consistent with the capacity of these T cells to home to inflammatory tissues (54). Then, we analyzed the expression of LFA-1 integrin, formed by the α-chain CD11a and the β-chain CD18, which is a major marker of adhesion and transmigration of T cells (55). Semen CD8^+^ T cells displayed high expression of this integrin, with 100% of the cells positive for CD11a and a mean of 40.3% ± 24.6 LFA-1^+^ cells. SIV infection did not significantly increase the proportion of LFA-1+ cells or CD11a+ CD18- cells (Mann-Whitney test, *p* = 0.38 and *p* = 0.79, respectively) (Figure 2H).

**Figure 3:**
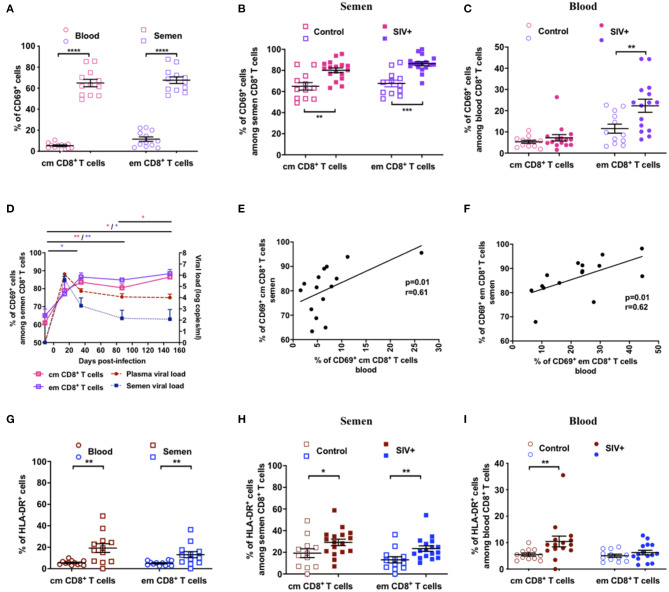
Activation profile of CD8^+^ T cells in semen and blood. **(A)** Comparison of CD69 expression on CD8^+^ T cells (Tcm: dark pink symbols, Tem: lavender symbols) in the blood (circles, *n* = 12) and semen (squares, *n* = 12) of uninfected macaques. **(B,C)** percentage of Tcm and Tem CD69^+^ CD8^+^ T cells in SIV^−^ (open symbols) and SIV^+^ (at > 1 year post-infection, filled symbols) macaques in semen **(B)** and blood **(C)**. **(D)** Longitudinal follow-up of the percentage of Tcm (dark pink line) and Tem (lavender line) CD69^+^ CD8^+^ T cells and of BVL (red dashed line) and SVL (blue dotted line) in eight macaques infected with 5,000 AID50 IV. The graph shows only viral loads data of animals for which CD69 results are available. Significant differences from baseline are shown (Wilcoxon matched-pairs signed rank test, *p < 0.05). **(E,F)** Spearman correlation between the percentage of Tcm **(E)** and Tem **(F)** CD69^+^ CD8^+^ T cells in blood and semen of 15 chronically infected macaques. **(G)** Comparison of HLA-DR **(B)** expression on CD8^+^ T cells (Tcm: brown symbols, Tem: blue symbols) in the blood (circles, *n* = 12) and semen (squares, *n* = 12) of uninfected macaques. **(H,I)** The percentage of Tcm (brown symbols) and Tem (blue symbols) HLA-DR^+^ CD8^+^ T cells in SIV^−^ (open symbols) and SIV^+^ (filled symbols) macaques at > 1 year post-infection in semen **(H)** and blood **(I)**. The mean and SEM are shown. *indicates significant differences between groups (Mann-Whitney test, *p < 0.05, **p < 0.01, ***p < 0.001, and ****p < 0.0001).

We further characterized Tcm and Tem CD8^+^ T cells by analyzing the expression of CD69 and HLA-DR, two T-cell activation markers that are commonly elevated on CD8^+^ T cells during HIV/SIV infection. In uninfected macaques, the percentage of CD69^+^ CD8^+^ T cells was much higher in the semen than blood (12- and of 6-fold higher for the Tcm and Tem subsets, respectively; Figure 3A), indicating a preexisting high level of activated T cells in the MGT. During infection, the proportion of CD69^+^ cells increased in the semen of SIV^+^ animals for both the Tcm (Mann-Whitney test, *p* = 0.002) and Tem (*p* = 0.0001) subsets (Figure 3B), whereas it was higher in the blood for only Tem cells (Mann-Whitney test, *p* = 0.0061, Figure 3C). Such a durable increase in the proportion of CD69^+^ cells was confirmed in eight infected macaques that we followed longitudinally up to 150 dpi (Figure 3D). Moreover, the proportion of CD69^+^ cells in both Tcm and Tem CD8^+^ cells correlated between blood and semen (Figures 3E,F). The percentage of HLA-DR^+^ CD8^+^ T cells was also higher in the semen than blood (4- and 3-fold for the Tcm and Tem subsets, respectively; Mann-Whitney test, *p* = 0.003 and *p* = 0.006, respectively, Figure 3G), confirming the activation state of semen cells. Following SIV infection, the proportion of CD8+ HLA-DR+ cells significantly increased in semen in both Tcm and Tem (Mann-Whitney test, *p* = 0.05 and 0.006, respectively, Figure 3H), whereas in blood the increase was observed only in the Tcm subset (Mann-Whitney test, *p* = 0.005, Figure 3I).

Thus, at steady state, semen CD8^+^ T cells display an activated memory profile typical of mucosal T cells (56, 57). SIV infection induces considerable changes in their phenotype, as well as in their dynamics. We then studied the SIV-specific CD8^+^ T-cell response to obtain a better insight into their function.

**Figure 4:**
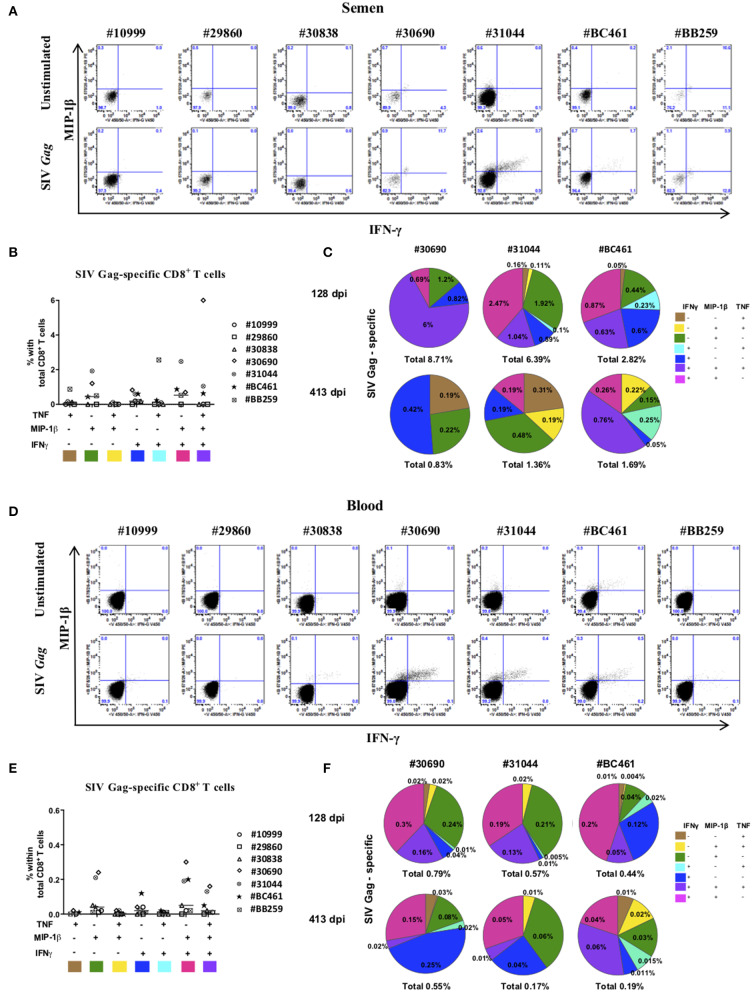
SIV-specific CD8^+^ T cells response in semen of SIVmac251-infected macaques. Intracellular staining for Th1 cytokines in semen CD8^+^ T cells in response to a 5-h stimulation with SIV *gag* peptide or without stimulation (unstimulated). **(A,D)** IFN-γ and MIP-ß expression in seminal plasma **(A)** and blood **(D)** CD8^+^ T cells of all studied animals (*n* = 7), with (lower panels) or without (upper panels) stimulation with *gag*. **(B,E)** Polyfunctionality of semen **(B)** and blood **(E)** CD8^+^ T cells in seven chronically infected macaques. IL-2 was not included because it was not produced in sufficient amounts. Each color represents one animal and the mean is shown. Shown are results after subtraction of results for the non-stimulated cultures. **(C,F)** Pie charts showing the polyfunctionality of semen CD8^+^ T cells in seminal plasma **(C)** and blood **(F)** of chronically-infected macaques with SIV-specific CD8 T cells (#30690, #31044, and #BC461) at steady state (128 dpi, upper panel) and the late chronic stage (413 dpi, lower panel). Macaques were infected with 5,000 AID50 IV.

To analyze the subset of T cells that recognized SIV Gag, we performed a 5-h *ex vivo* stimulation experiment with a pool of SIV *Gag* peptides on semen cells and PBMCs to assess and quantify the presence of SIV-specific CD8^+^ T cells and assessed the expression of IFN-γ, TNF, IL-2, and MIP-1β (Figures 4A,D). There was no significant expression of IL-2, neither in cells from the blood or semen (not shown). There was a clear SIV-specific CD8^+^ T-cell response at 128 dpi for three of seven macaques studied (#31044, #30690, and #BC461) in both seminal plasma (Figures 4B,C) and blood (Figures 4E,F). In seminal plasma, we observed four types of SIV-specific cytokine-producing CD8^+^ T cells: mostly highly polyfunctional IFN-γ^+^MIP-1β^+^TNF^+^ cells (6.0, 1.0, and 0.6% positive cells for #30690, #31044, and #BC461, respectively), followed by IFN-γ^−^MIP-1β^+^TNF^−^ (1.2, 1.9, and 0.4% positive cells for #30690, #31044, and #BC461, respectively), IFN-γ^+^MIP-1β^+^TNF^−^ (0.7, 2.5, and 0.9% positive cells for #30690, #31044, and #BC461, respectively), and IFN-γ^+^MIP-1β^−^TNF^−^ cells (0.8, 0.6, and 0.6% positive cells for #30690, #31044, and #BC461, respectively) (Figures 4B,C). The SIV-specific response of all these macaques displayed the same dominant profiles in blood (Figures 4E,F): mostly IFN-γ^+^MIP-1β^+^TNF^−^ cells (0.3, 0.2, and 0.2% positive cells for #30690, #31044, and #BC461, respectively), followed by IFN-γ^−^MIP-1β^+^TNF^−^ (0.2, 0.2, and 0.04% positive cells for #30690, #31044, and #BC461, respectively), and highly polyfunctional IFN-γ^+^MIP-1β^+^TNF^+^ cells (0.2, 0.1, and 0.05% positive cells for #30690, #31044, and #BC461, respectively). The proportion of these SIV-specific cells was much lower in blood than in semen. We still detected a polyfunctional SIV-specific response in semen CD8^+^ T cells of animals #31044 and #BC461 at a late stage of infection (413 dpi), although at a lower proportion (Figure 4C). The proportion of IFN-γ^+^, TNF^+^, and MIP-1β^+^ cells in neither compartment (blood or semen) correlated with the control of viral RNA loads (data not shown).

In conclusion, most of the SIV-specific cells of both compartments displayed high polyfunctionality, with concomitant expression of at least two of the three cytokines IFN-γ, MIP-1β, and TNF. The SIV-specific response we observed in semen CD8^+^ T cells was not associated with reduced SIV shedding. However, in the semen, the macaques with the highest SIV-specific responses in CD8^+^ T cells displayed the highest SVL (data not shown). The same trend was observed in blood, with the exception of one macaque (#30742), which had the highest proportion of cells positive for the three cytokines and the lowest BVL (data not shown).

### Analysis of SIV-Specific Antibody Titer and Functions in the Semen Reveals a Role for ADCC in the Modulation of Semen Infectivity

**Figure 5:**
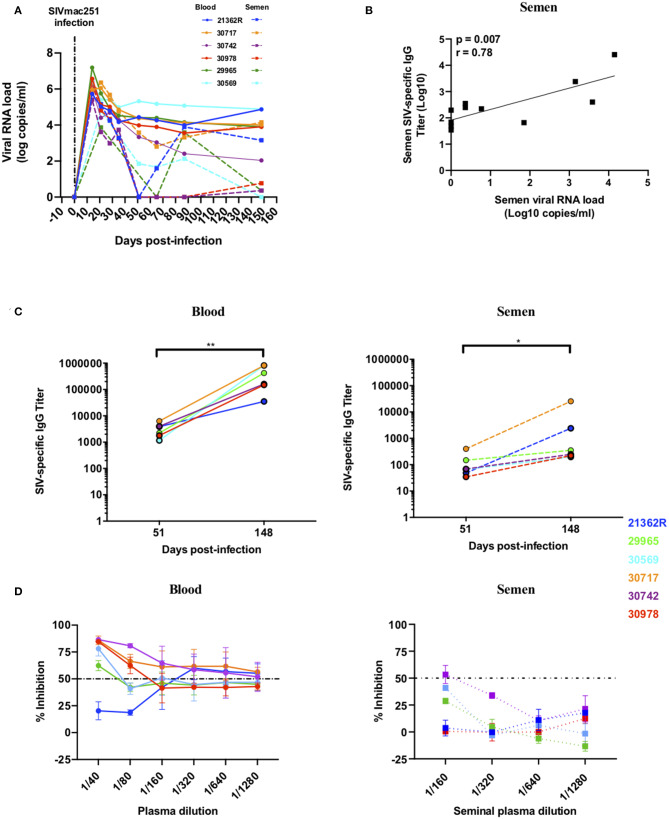
Viral loads, anti-SIV IgG titers and neutralizing antibody response in semen and blood of SIVmac251 infected macaques. Longitudinal follow-up of viral loads, IgG targeting SIV, and neutralizing Ab response in six infected macaques. Each color represents a different animal. **(A)** Blood and seminal plasma viral RNA loads (BVL: solid lines, SVL: dotted lines) of six macaques infected with 5,000 AID50 IV. **(B)** Spearman correlation between semen SIV-specific IgG titers and SVL at 51 and 148 dpi. **(C)** Blood (left panel) and seminal plasma (right panel) titers of SIV-specific IgG for each macaque (the 2 titer values are connected by a line) at baseline and 51 and 148 dpi. **(D)** Neutralizing antibody response in the blood (left panel) and seminal plasma (right panel) of six infected macaques at 148 dpi. The dotted line represents 50% of viral inhibition. The mean and SEM are shown. *p < 0.05 and **p < 0.01.

The presence of Abs with neutralization activity or those able to activate Fc-mediated functions could have a large impact on the infectivity of semen. We therefore analyzed these functions in paired blood and semen samples from six macaques longitudinally followed up to 148 dpi (Figure 5A). Interestingly, a strong positive correlation was observed between SIV-specific Ab titers and viral load in semen (*r* = 0.78, *p* = 0.007) but not blood (Figure 5B and data not shown), suggesting that there may be local Ab response in response to virus replication. The profile of SIV-specific Ab titers among individuals was highly diverse (Figure 5C). Ab titers were detected at low levels 51 dpi in blood and semen (mean titer of 3223 ± 1890 and 128 ± 139, respectively) and increased at the chronic stage (148 dpi) in both compartments (mean titer of 401344 ± 347696 and 4836 ± 10209 in blood and semen respectively; Figure 5C). Titers at 148 dpi were significantly higher than at 58 dpi (Mann-Whitney test, *p* = 0.002 and *p* = 0.03 in blood and semen, respectively) and titers in blood were significantly higher than in semen at both time points (Mann-Whitney test, *p* = 0.002 at both 51 dpi and 148 dpi). During the chronic stage, the most elevated titers in the seminal plasma were observed for two macaques (#30717 and #21362R) that displayed constant high viral shedding in blood and semen (“high shedder”) (Figures 5A,C). A correlation between anti-SIV specific IgG titers in the two compartments was not observed (*r* = 0.48, *p* = 0.12, data not shown). It is possible that such specific responses can neutralize SIV, since neutralizing Abs (NAbs) produced in the MGT may modulate the per-act risk of sexual transmission. We assessed the NAb response against the challenge virus SIVmac251, a tier 2 virus, using blood serum and seminal plasma at 148 dpi. Seminal plasma is known to have a toxic effect on cell. Thus, we performed preliminary experiments to assess semen toxicity on TZM-bl cells (data not shown). Based on the results obtained, we used semen at a starting dilution of 1/160 (vs. 1/40 used to assay blood serum) to avoid nonspecific inhibition and/or cell death. We detected NAbs in the blood of five of the six macaques and in the seminal plasma of only one (#30742) (Figure 5D). The responses were generally weak in both compartments, but specific, as assessed using a VSV-pseudotyped virus as a control (data not shown).

**Figure 6:**
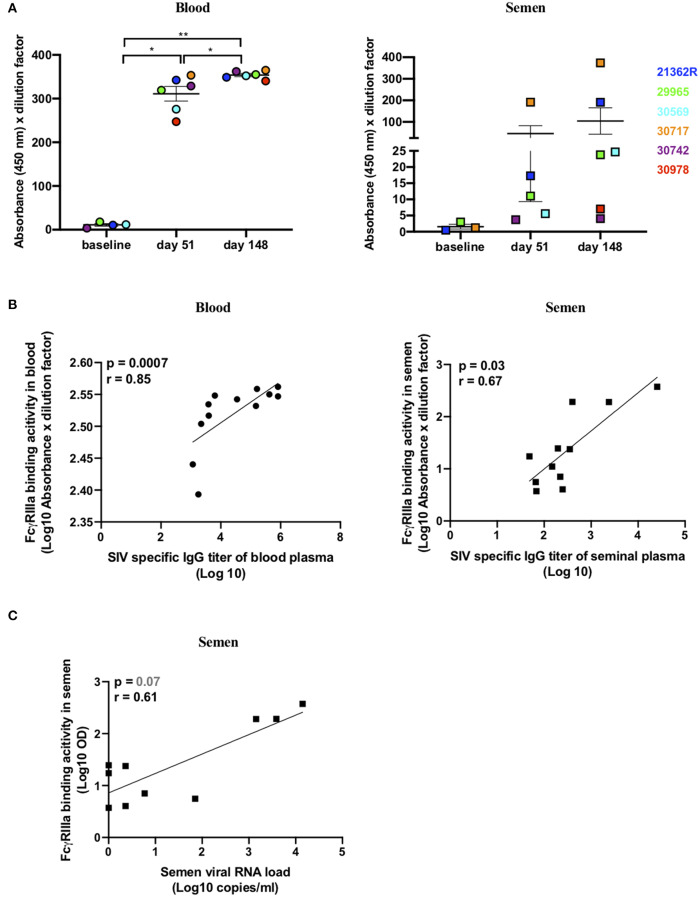
SIV-specific dimeric rsFcγRIIIa binding antibodies in paired blood and semen samples of SIVmac251 infected macaques. **(A)** FcγRIIIa-V158 binding Ab response in blood (left panel) and seminal plasma (right panel) to SIVmac251 gp130 protein. Results are presented as absorbance at 450 nm multiplied by the dilution factor (1/100) of each sample. The mean and SEM are shown, and each color represents a different animal. The responses at 51 and 148 dpi were compared using the paired *t* test. *p < 0.05 and **p < 0.01 was considered significant. **(B)** Spearman correlation between SIV-specific Ab titers and the FcγRIIIa -V158 binding Ab response in blood (left panel) and seminal plasma (right panel). **(C)** Spearman correlation between FcγRIIIa -V158 binding Ab response and SVL.

As the NAb response was very weak, we then sought to evaluate whether Abs with ADCC functions were present in the seminal plasma samples. The cross-linking of FcγRs by Abs is a process essential to initiate ADCC responses. A novel ELISA-based IgG assay, using a soluble recombinant FcγRIIIa dimeric protein, recapitulates this process *in vitro* (44, 58, 59). As previously described, recombinant FcγR engagement by Abs correlates with measures of functional ADCC using cell-based assays (44, 58). Thus, we assessed the ability of SIVmac gp130-bound Abs present in the blood and semen of infected macaques to engage soluble recombinant FcγRIIIa as a surrogate of ADCC activity. Abs able to engage FcγRIIIa emerged at 51 dpi in the blood of six/six macaques relative to baseline values (mean absorbance ± SD = 11 ± 5.9) (paired *t* test, *p* = 0.04) and significantly increased by 148 dpi (*p* = 0.004) (Figure 6A). FcγRIIIa-binding Abs were present in the semen of three/six animals at 51 dpi, relative to baseline values (mean absorbance ± SD = 1.5 ± 1.3), at high titers in macaque #30717 and at low titers in animals #21362R and #29965. By 148 dpi, the titers increased in the three macaques and FcγRIIIa-binding Abs became detectable in a fourth animal (#30569) (Figure 6A). FcγRIIIa-binding Abs correlated with total anti-SIV Ab titers in both blood and semen (*r* = 0.85, *p* = 0.0007 in blood and *r* = 0.67, *p* = 0.03 in semen; Figure 6B). Unexpectedly, a trend toward a positive association between viral load and FcγRIIIa-binding Ab titers in the semen was observed (*r* = 0.61, *p* = 0.07) (Figure 6C). On the contrary, there was no correlation in the systemic compartment (*r* = −0.23, *p* = 0.4708 data not shown). Indeed, the two “high shedder” animals (#30717 and #21362R) also had the highest semen FcγRIIIa-binding Ab titers (Figures 5A,B). This suggests that continuous antigen stimulation is necessary to evoke Abs with a potential ADCC function.

## Discussion

HIV/SIV infection is associated with increased level of cytokines in the periphery and some of them are even more elevated in the seminal plasma of infected individuals (23, 40, 60–65). Here, we report the first extensive study evaluating the effect of SIV infection on the seminal cytokine/chemokine network. Many molecules correlate with BVL and/or SVL, including pro-inflammatory molecules (Il-1β, IL-8, IL-15, IL-18, RANTES) and immunomodulatory cytokines (IL-13 and TGF-β). These molecules are known to modulate immune-cell activation and migration profiles, as well as to initiate T-cell effector functions (66). They may both influence the leukocytes that are present in the donor's semen and the partner's mucosal tissue, thereby influencing the efficacy of viral transmission. For example, the immunosuppressive properties of TGF-β could be detrimental in the context of sexual transmission of HIV-1 because it would divert cell-mediated immune responses. When deposited and activated in the female reproductive tract (FRT), TGF-β present in semen elicits a transient pro-inflammatory response, with the consequent influx and activation of macrophages, dendritic cells, and neutrophils (67). Our results further confirm and extend previous reports (60–62) that SIV infection in the MGT has a profound effect on semen composition, leading to altered cytokine profiles that can modulate HIV/SIV replication, promote viral shedding, and cause local target-cell activation. Moreover, these observations highlight the need of HIV-1 vaccine studies to consider the potential impact of semen exposure on the mucosal immune system.

The highly inflammatory environment we describe in the semen of SIV-infected macaques possibly mirrors what happens in the MGT and can potentially modulate the activation status of immune cells. Indeed, semen CD8^+^ T cells displayed an activation profile, with a higher proportion of CD69^+^ and HLA-DR^+^ cells than in peripheral blood. In addition, the proportion of CD69^+^ cells was higher in SIV-infected macaques in both compartments, particularly in effector cells, consistent with the results of previous studies conducted on HIV^+^ individuals (68) and SIV^+^ macaques (69). Furthermore, in this study, the proportion of CD69^+^ central memory and effector memory CD8^+^ T cells in the blood correlated with that in the semen of SIV^+^ macaques, indicative of generalized immune activation in SIV-infected macaques and suggesting that systemic immune activation could be used to predict levels in the genital tract. The activation state of CD8+ T cells is also confirmed by a higher expression of HLA-DR by both the central memory and effector memory subset in semen compared to blood, which further increased following SIV infection. SIV-infected macaque CD8^+^ T cells displayed high proportions of CCR5^+^ CXCR3^−^ and CCR5^+^CXCR3^+^ cells, a phenotype associated with the inflammatory homing Tc1 effector profile (51, 52). The increased expression of CXCR3^+^ is paralleled by high levels of it's ligand IP-10, supporting a role for the chemokine in effector cells recruitment to mucosal sites. Altogether, these observations strongly suggest that semen CD8^+^ T cells are of mucosal origin and undergo substantial modifications in their activation profile and effector function in infected animals. The role of such effector T cells in the semen and whether their excretion is active or passive are still unclear. Interestingly, activated, cytotoxic (TIA-1^+^) CD3^+^ T-cell infiltrates have been reported along the MGT (mostly in the prostate, seminal vesicles, and epididymis) of SIV-infected macaques displaying high viremia (70). Semen CD8^+^ T cells may originate from these cellular infiltrates of the various semen-producing organs and therefore reflect the inflammatory status within the MGT.

Cell-mediated immunity plays a major role in controlling HIV-1 replication in humans and SIV replication in macaques (18, 40). Moreover, the presence of highly polyfunctional T cells has been reported in elite controllers and long-term non-progressor HIV patients (24, 71). Here, we investigated the SIV-specific CD8^+^ T-cell response in semen and its potential role in HIV/SIV transmission. The three animals that displayed a detectable SIV-specific T-cell response had a high proportion of IFN-γ, TNF and MIP-1β producing cells and mostly polyfunctional cells. Remarkably, these responses were approximately 10-fold higher in semen than peripheral blood. Importantly, such a high SIV-specific response in semen was associated with elevated local viral shedding. Although the numbers of animals in this study with SIV Gag-specific T cell responses is small, this suggests that the SIV-specific T cells in semen do not control replication of the virus. Moreover, although the modest numbers did not allow statistical analysis nor the possibility to draw specific conclusions, some points warrant discussion. The CD8^+^ T cell response in the blood during acute HIV or SIV infection increased following the increase in viral load and there was an inverse correlation between viral load and the CD8^+^ T cell response during primary infection (72–74). High CD4^+^ T-cell and CD8^+^ T cell IFN-γ responses during early infection in Cynomolgus macaques were associated with better long-term viral control (39). During chronic infection, polyfunctional HIV-specific CD8^+^ T-cell responses were maintained in non-progressors (24). The importance of CD8^+^ T-cell responses in controlling viral replication was shown a long time ago by *in vivo* depletion of CD8^+^ T cells in SIV-infected macaques (75, 76). In our cross-sectional study, we did not observe the same profile in the semen. On the contrary, the CD8^+^ T-cell effector function appeared to have no effect on local viral shedding. Although these studies are technically demanding due to accessing semen and the modest numbers of cells recovered, future studies could analyze responses to a broader range of SIV proteins to understand the breadth of the T cell immunity.

Humoral immunity in semen is likely to be important in HIV/SIV transmission. We found that the dynamics of viral RNA loads and SIV-specific Abs in the blood and semen of SIVmac251-infected macaques were similar to those reported for humans (6, 45, 47). SIV-specific Abs were detected in all semen samples, generally at lower levels than in serum, confirming the observations previously reported for HIV-1 infected subjects (77). While a positive association was observed between seminal viral load and semen IgG titers, such a correlation was not observed in blood, suggesting that local humoral response failed to control viral shedding. This is consistent with observations made in human patients, whose systemic antibody response to HIV following infection is mostly ineffective, with an early B-cell response being largely directed against non-neutralizing epitopes of the HIV envelope. The response during late stages is impaired by aberrant B-cell hyperactivation (78–80). We did not find a correlation between IgG titers in the two compartments, suggesting local antibody production. The origin of antibodies in the MGT is controversial, with one study suggesting local synthesis of IgG (81) and another indicating a blood origin of semen antibodies (82). It is possible that technical differences account for the disparate results. Moldoveanu et al. reported that seminal plasma contains plasma derived IgG to systemically injected vaccines and naturally occurring secretory IgA to environmental antigens of microbial origin and to an orally administered bacterial vaccine (83). Thus, from the limited studies of naturally occurring and immunization-induced specific antibodies, it appears that secretions of the male genital tract contain IgG of both local and systemic origin (26, 84) while at least a fraction of IgA is locally produced by the glands of Littré distributed along the penile urethra (85). Studies are instead concordant in the conclusion that in seminal plasma, IgG not IgA is the predominant Ig isotype, and that IgM is present at low level (26, 83). The limited amount of seminal plasma did not allow us to investigate the presence of IgA or IgM, nevertheless, future studies should evaluate subclasses of antibodies in the semen of macaques to address similarities or differences to human semen.

Here, we report the presence of Abs that neutralize the autologous virus in five of six serum samples and in one of six semen samples from macaques at 148 dpi. Thus, NAbs seem to develop more easily at the systemic level than at the mucosal site. The only macaque (#30742) with a weak NAbs response in semen also had the strongest neutralizing response in serum. Both serum and semen from these macaques did not inhibit infection by a VSV-pseudotyped virus, indicating that although weak, the observed response was specific. SIVmac251 is a “difficult to neutralize” virus, so the lack of neutralization observed is not surprising. However, we cannot exclude that the detection of NAbs in the semen may have been underestimated and that semen dilutions below 1/160 could inhibit infection.

Although a protective role against HIV-1 acquisition has been reported for Abs present in genital secretions, env-specific IgG present in semen may instead facilitate mucosal transmission of HIV-1. Indeed, a proportion of HIV virions may be coated with IgG in semen and form an immune complex that can cross the mucosal epithelium, as previously reported for anti-HIV broadly NAbs (86). The presence of Abs in genital secretions, such as semen, should be considered in the design of prevention strategies, as it could impede attempts to provide immune-based prophylactics and/or vaccines. However, we cannot exclude that the efficiency of vaccine-induced or passively transferred Abs might be reduced in prophylactic approaches by the presence of virion-Ig complexes.

Studies have reported the presence of Abs with ADCC activity in cervico-vaginal fluids from HIV-1 infected women with lower genital viral load (87, 88), in HIV controllers (89) and in chronically HIV-1-infected individuals who did not transmit infection to their heterosexual ESN partners (90). However, little is known about ADCC activity in the MGT and Fc-mediated antibody functions have been mostly overlooked in semen. Only one study reported the presence of ADCC Abs in the semen of HIV-1-infected individuals (38). The presence of ADCC-mediating Abs has not been previously evaluated in the semen of macaques infected with SIV. Therefore, we evaluated the presence of FcγRIIIa dimeric protein-binding semen Abs in infected macaques as a surrogate of ADCC function and investigated whether it may be associated with local viral shedding. We detected Abs that engage the FcγRIIIa in the blood of all of the studied macaques. The responses were weaker in the semen, but detectable in four of six animals. Although we did not find a correlation between the presence of Abs that bind the dimeric FcγRIIIa and BVL, there was a positive correlation in the semen. Indeed, the two macaques with the highest FcγR-binding titers were those with the highest SVL at 5 months post-infection. These results need to be confirmed in a larger cohort. Although our results suggest that the ADCC response does not protect against viral shedding nor disease progression, they do suggest that Abs with a potential to mediate ADCC could modulate semen infectivity and viral transmission. As reported (38), the presence of ADCC Abs in semen may, at least in part, explain the relatively low rate of sexual transmission during sexual intercourse.

The limited amount of available seminal plasma did not allow us to investigate other Fc-mediated antibody functions, such as antibody-mediated viral inhibition (ADVI) and antibody-mediated phagocytosis (ADP), which may also contribute to protection from HIV infection. Future studies should investigate these properties, as ADCVI has been observed with mucosal anti-HIV-1 IgG in the genital tract of HIV-infected women (91) and because it can explain, at least in part, the protective effect of a vaccine against the acquisition of neutralization-resistant SIV challenge in Rhesus macaques (92). Furthermore, studies investigating the contribution of the biophysical properties of ADCC Abs present in semen, such as epitope avidity, epitope specificity, and IgG subclass distribution, will be of interest, as these functions have been shown to be associated with the control of disease and protection in vaccine studies (93, 94).

Overall, this comprehensive study found that semen from SIV infected macaques contained high levels of pro-inflammatory cytokines and chemokines and, in contrast to the blood, was enriched for activated CD8^+^ memory T cells. Thus, responses observed in the periphery do not reflect the local immune response and inflammatory features of the MGT response, which could enhance transmission via effects on the functional status of either the seminal leukocytes or the recipient mucosa. High seminal viremia was associated with mucosal IgG responses with potential ADCC function. Identifying and optimizing the protective efficacy of immune responses in the MGT may be key to achieving vaccine-induced immunity to sexually transmitted HIV-1 infection.

## Data Availability Statement

The datasets generated for this study are available on request to the corresponding author.

## Ethics Statement

The animal study was reviewed and approved by Comité d'Ethique en Experimentation Animale de la Direction des Sciences du Vivant au CEA.

## Author Contributions

RL and MC: study conception and design. KS, SB-S, CG, and BD: acquisition of data. KS, SB-S, CG, ND-B, and MC: analysis and interpretation of data. KS and MC: drafting of the manuscript. KS, SK, BW, PH, RL, and MC: critical revisions. SK, BW, and PH: contributed with reagents and expertise.

## Conflict of Interest

The authors declare that the research was conducted in the absence of any commercial or financial relationships that could be construed as a potential conflict of interest.
